# Transcriptome-wide 5-methylcytosine modification profiling of long non-coding RNAs in A549 cells infected with H1N1 influenza A virus

**DOI:** 10.1186/s12864-023-09432-z

**Published:** 2023-06-12

**Authors:** Shengqiang Jiang, Jing Hu, Yang Bai, Ruiwei Hao, Long Liu, Hongying Chen

**Affiliations:** 1 College of Life Sciences, Northwest A & F University, Yangling, 712100 Shanxi P. R. China; 2 School of Basic Medical Sciences, Hubei University of Medicine, Shiyan, 442000 Hubei P. R. China

**Keywords:** Influenza virus (IAV), Long noncoding RNA (lncRNA), 5-methylcytosine (m^5^C) modification, Methylated RNA immunoprecipitation sequencing (MeRIP-Seq), A549 cells

## Abstract

**Background:**

In recent years, accumulating evidences have revealed that influenza A virus (IAV) infections induce significant differential expression of host long noncoding RNAs (lncRNAs), some of which play important roles in the regulation of virus-host interactions and determining the virus pathogenesis. However, whether these lncRNAs bear post-translational modifications and how their differential expression is regulated remain largely unknown. In this study, the transcriptome-wide 5-methylcytosine (m^5^C) modification of lncRNAs in A549 cells infected with an H1N1 influenza A virus was analyzed and compared with uninfected cells by Methylated RNA immunoprecipitation sequencing (MeRIP-Seq).

**Results:**

Our data identified 1317 upregulated m^5^C peaks and 1667 downregulated peaks in the H1N1 infected group. Gene ontology (GO) and the Kyoto Encyclopedia of Genes and Genomes (KEGG) analyses showed that the differentially modified lncRNAs were associated with protein modification, organelle localization, nuclear export and other biological processes. Furthermore, conjoint analysis of the differentially modified (DM) and differentially expressed (DE) lncRNAs identified 143 ‘hyper-up’, 81 ‘hypo-up’, 6 ‘hypo-down’ and 4 ‘hyper-down’ lncRNAs. GO and KEGG analyses revealed that these DM and DE lncRNAs were predominantly associated with pathogen recognition and disease pathogenesis pathways, indicating that m^5^C modifications could play an important role in the regulation of host response to IAV replication by modulating the expression and/or stability of lncRNAs.

**Conclusion:**

This study presented the first m^5^C modification profile of lncRNAs in A549 cells infected with IAV and demonstrated a significant alteration of m^5^C modifications on host lncRNAs upon IAV infection. These data could give a reference to future researches on the roles of m^5^C methylation in virus infection.

**Supplementary Information:**

The online version contains supplementary material available at 10.1186/s12864-023-09432-z.

## Background

RNAs are subject to a range of covalent modifications at the single nucleotide level, such as pseudouridine, N6-methyladenosine (m^6^A) [1], N1-methyladenosine (m^1^A) [2], 5-methylcytosine (m^5^C) [3]. So far, over 100 distinct RNA modifications have been described. As a widespread modification of RNA, 5-methylcytosine (m^5^C) has received considerable attention in recent years. Increasing numbers of studies have demonstrated that RNA m^5^C modification plays important roles in multiple biological processes including RNA processing [4, 5], RNA stability [6, 7], RNA transport [8], and mRNA translation [9]. With the development of high-throughput sequencing, it is now possible to identify and quantify m^5^C modifications in low-abundance RNA species such as non-coding RNAs (ncRNAs), and transcriptome-wide identifications of m^5^C modification in different types of RNAs have been reported [10–12].

Evidence is emerging that RNA modifications are involved in the regulation of virus infection. Epitranscriptomic marks such as m^6^A, m^5^C, N4-acetylcytidine (ac^4^C) and 2ʹO-methylated nucleosides (Nm) have been reported to promote viral replication by upregulating viral mRNA stability or translation, or by preventing the recognition of viral RNA through modulation of host RNA-specific innate immunity factors including RIG-I and MDA5 [13]. As a relatively common epitranscriptomic mRNA modification, m^5^C has been found to present at higher levels in retroviral transcripts than in cellular mRNAs, and the modification can regulate RNA splicing and promote the translation of viral mRNAs [14]. A more recent study has demonstrated that RNA m^5^C methylation can control antiviral innate immunity through modulating the m^5^C methylome of noncoding RNAs (ncRNAs) and their expression, which regulate type I interferons [15].

As an important pathogen for seasonal respiratory illness, influenza A viruses (IAV) have caused human pandemic outbreaks such as those occurred in 1918, 1957 and 1968, and they are still continuing to threaten public health [16, 17]. IAV primarily infects respiratory epithelial cells and causes pulmonary diseases. If uncontrolled, the infection can cause loss of lung function and even mortality [18, 19]. Understanding the process of IAV infection is critical to target IAV-induced pathogenesis and develop effective anti-viral approaches. Recent studies have revealed that host long noncoding RNAs (lncRNAs) are key regulators of host-virus interactions during viral infection [20, 21]. Thousands of lncRNAs have been identified to be differentially expressed during IAV infection [22–24]. Some of them affect IAV infection by regulating the host innate immune responses [25–30], modulating cellular metabolism [31] or directly interacting with viral proteins [32, 33].

Epitranscriptomic regulation has been shown as an important mechanism to control lncRNAs expression and tissue specificity [31]. Although m^5^C is a common epitranscriptomic modification found in RNAs, knowledge surrounding the prevalence and transcriptome-wide distribution of m^5^C in lncRNA is still very limited, and the roles of m^5^C modification during IAV infection have not yet been explored. It remains unknown whether m^5^C modification plays a role in regulating lncRNA expression during IAV infection.

In this study, the m^5^C methylation of lncRNAs in H1N1-infected A549 cells were globally mapped by Methylated RNA immunoprecipitation sequencing (MeRIP-seq), using the uninfected cells as controls. Marked alterations in the amount and distribution of m^5^C peaks in lncRNAs were detected between the two groups, suggesting that m^5^C modifications could play important regulatory roles during IAV replication.

## Results

### Differentially expressed lncRNAs in H1N1 infected and uninfected A549 cells

**Fig. 1:**
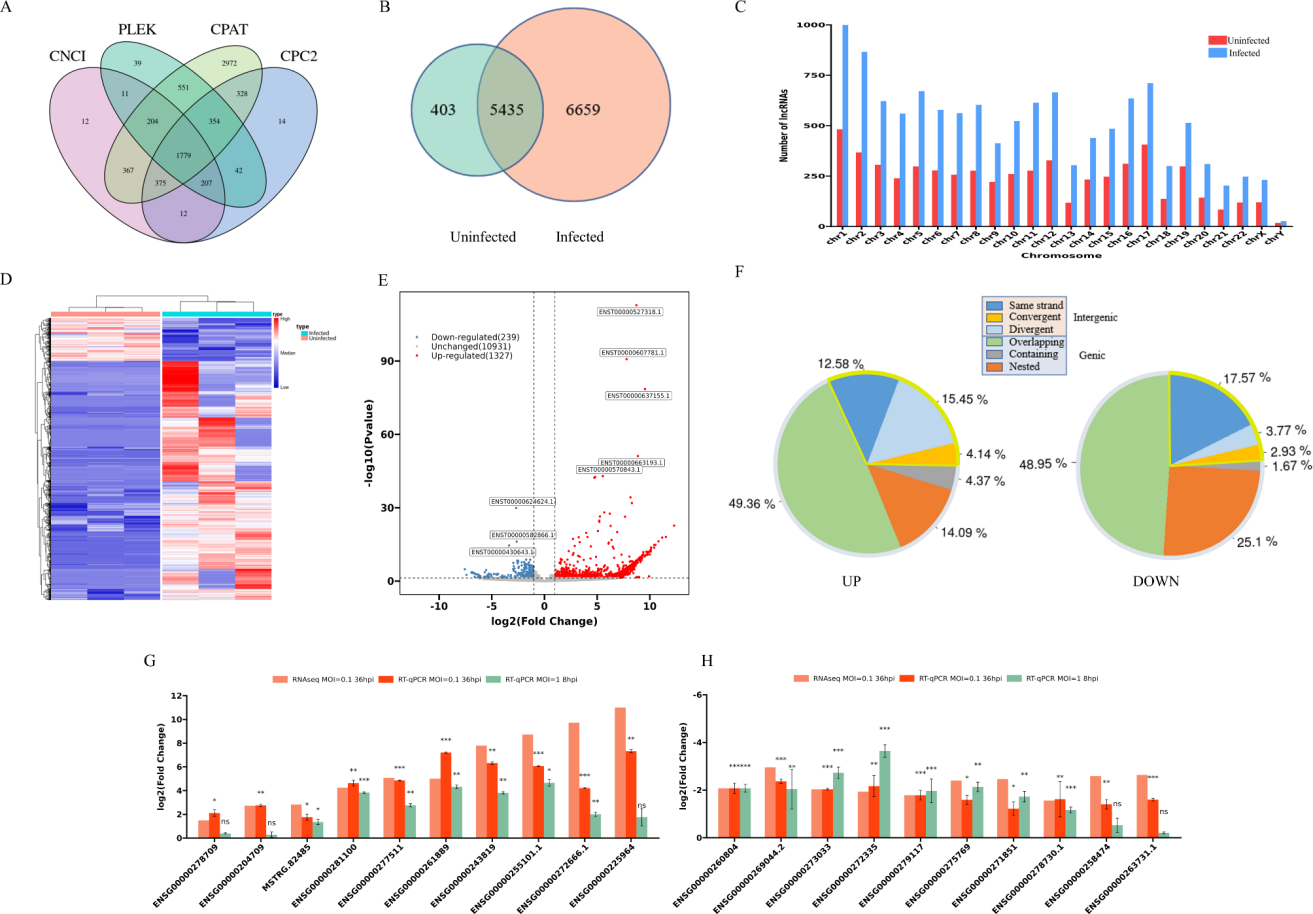
Expression profile of lncRNAs in H1N1-infected and uninfected A549 cells. **(A)** Venn diagram of the coding potential prediction results for novel lncRNAs by four analysis tools (CPC2, PLEK, CNCI, and CPAT). Those lncRNAs with no predicted protein-coding potential by all the four analysis tools were designated as novel lncRNAs and used for subsequent analysis. **(B)** The Venn diagram showing the numbers of lncRNAs identified in uninfected and H1N1-infected cells. **(C)** Distribution of lncRNAs on chromosomes in uninfected and H1N1-infected cells. **(D)** The heatmap showing the changes in lncRNAs expression levels upon IAV infection. **(E)** The volcano plot of the differentially expressed (DE) lncRNAs in uninfected and H1N1-infected cells. Red dots represent 1327 significantly up-regulated lncRNAs after H1N1 infection. Blue dots represent 239 significantly down-regulated lncRNAs after H1N1 infection. Grey dots indicate lncRNAs with no significant changes after H1N1 infection. The thresholds for screening significantly differentially expressed lncRNAs were P-value < 0.05, log_2_FC > 1 or < -1. **(F)** Classification of DE lncRNAs by FEELnc. **(G)** Verification of upregulated lncRNAs in transcriptome sequencing data by RT-qPCR. GAPDH was examined as an internal standard. A549 cells infected with IAV at MOI 0.1 were harvested at 36 hpi to verify the transcriptome sequencing data, and cells infected at MOI of 1 were harvested at 8hpi to examine the expression levels of lncRNAs in early infection. *: p < 0.05, **: p < 0.01, ***: p < 0.001, ns: not significant, in IAV-infected cells vs. uninfected cells. **(H)** Verification of downregulated lncRNAs in transcriptome sequencing data by RT-qPCR. GAPDH was examined as an internal standard. A549 cells infected with IAV at MOI 0.1 were harvested at 36 hpi to verify the transcriptome sequencing data, and cells infected at MOI of 1 were harvested at 8hpi to examine the expression levels of lncRNAs in early infection. *: p < 0.05, **: p < 0.01, ***: p < 0.001, ns: not significant, in IAV-infected cells vs. uninfected cells

RNAseq was performed to determine whether the lncRNA expression profile changed upon IAV infection. In total, 12,497 lncRNAs were detected in the IAV infected and uninfected A549 cells, including 1779 novel lncRNAs which were predicted as noncoding transcripts by all the four coding prediction softwares used in this study (Fig. 1A). Among the identified lncRNAs, 5435 expressed in both the infected and uninfected cells, 403 were uniquely detected in the uninfected group and 6659 lncRNAs were only detected in the H1N1-infected group, suggesting that H1N1 infection remarkably changed the expression profile of lncRNAs in the host cell (Fig. 1B). In both the infected and uninfected cells, the expressed lncRNAs were widely distributed across all chromosomes. The H1N1-infected group had a higher number of lncRNAs detected in all chromosomes than the uninfected group, demonstrating that the changes in lncRNA expression profile had taken place in all chromosomes (Fig. 1C).

Hierarchical clustering was conducted to analyze the lncRNA expression profile between the H1N1-infected and uninfected groups. Obviously, the expression levels had significantly altered upon H1N1 infection (Fig. 1D). The differential expression profile between the two groups was shown in Volcano plot in Fig. 1E. In total, there are 1566 lncRNAs that were significantly differentially expressed (DE), including 1327 up-regulated and 239 down-regulated lncRNAs (|FC|>2, P-value < 0.05) (Additional file 1).

LncRNAs are classified into intergenic and genic types which are further divided into six subtypes. Classification analysis showed that IAV infection did not dramatically change the proportional distribution of the different subtypes, and more than half of the DE lncRNAs were genic type (68%) and only 32% belonged to intergenic type (Supplementary Fig. 1 in Additional file 4). When the up-regulated and down-regulated lncRNAs were separately analyzed, it turned out that overlapping genic subtype accounted for about half of the DE transcripts in both groups. The ratios of nested genic subtype and same strand intergenic subtype were higher in the down-regulated lncRNAs than that in the up-regulated ones, while the other three subtypes, especially the divergent intergenic subtype, accounted for a larger proportion in the up-regulated lncRNAs than the down-regulated lncRNAs (Fig. 1F).

To verify the reliability of the RNA-Seq results, we selected 20 DE lncRNAs (10 upregulated and 10 downregulated) and detected their expression levels by RT-qPCR. In the A549 cells infected by 0.1 MOI of IAV at 36 hpi, the qPCR results were consistent with the RNA-Seq results (Fig. 1G-H). To evaluate whether the differential expression of these lncRNAs was the early response upon the virus infection, A549 cells infected by 1 MOI of IAV at 8 hpi were also examined by RT-qPCR (Fig. 1G-H), which identified the differential expression of 15 lncRNAs. The rest 5 lncRNAs showed consistent trend of upregulation or downregulation of expression at 8 hpi with that of 36 hpi, but their expression levels in IAV-infected cells were not significantly changed from the levels in uninfected cells, indicating that the differential expression of these lncRNAs at 36 hpi might be follow-up responses to other cellular factors that altered in early infection.

### Transcriptome-wide m^5^C methylation of lncRNAs in IAV infected cells

**Fig. 2:**
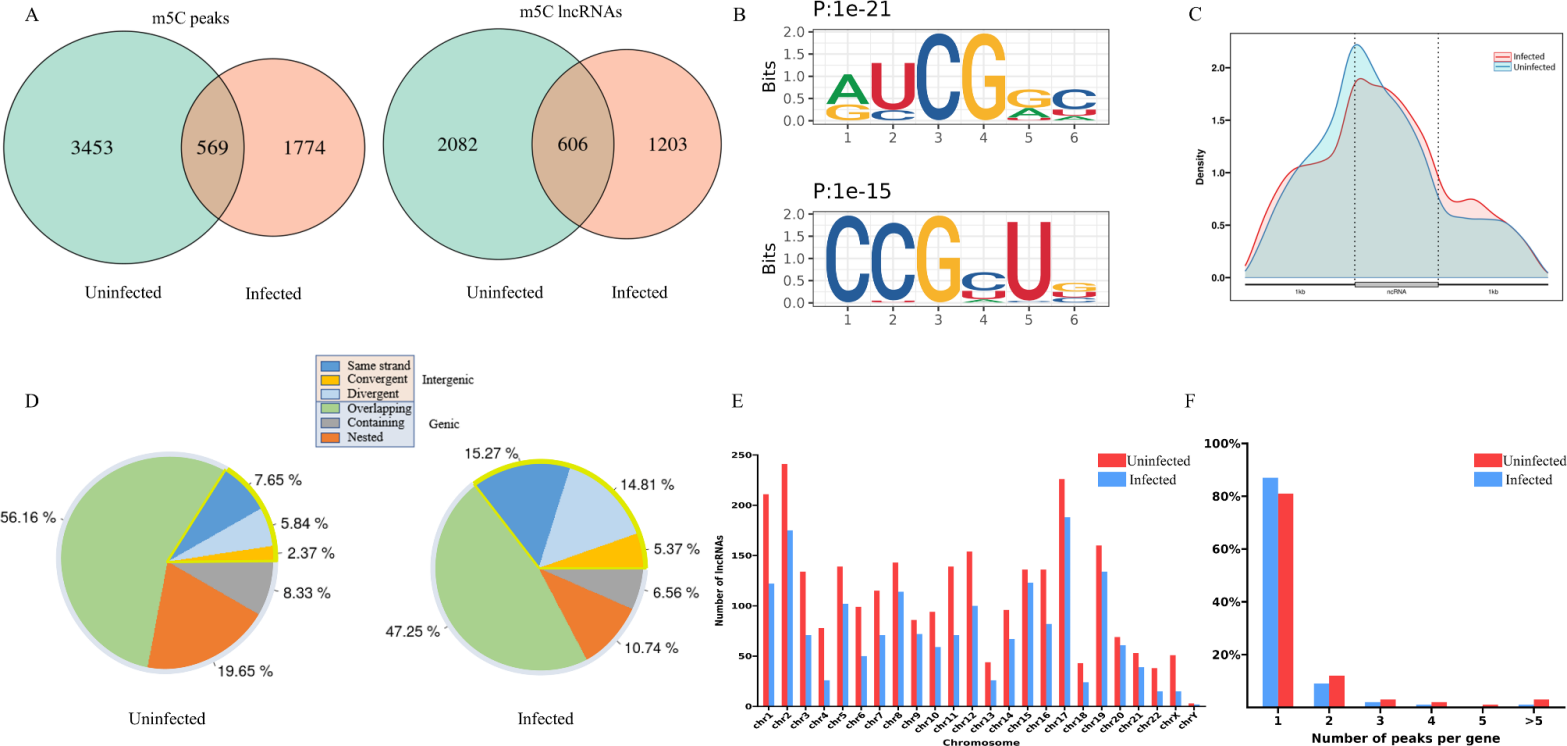
Landscape of m^5^C methylation on lncRNAs in uninfected and H1N1-infected cells. **(A)** Venn diagram showing the numbers of m^5^C peaks (left) and modified lncRNAs (right) in uninfected and H1N1-infected cells. **(B)** The two consensus motifs of m^5^C sites on lncRNAs. **(C)** Density distribution of m^5^C in lncRNA transcripts. **(D)** Classification of m^5^C modified lncRNAs by FEELnc in uninfected and H1N1-infected cells. **(E)** Distribution of modified lncRNAs on chromosomes in uninfected and H1N1-infected cells. **(F)** The proportion of lncRNAs with different number of m^5^C peaks in the two groups

To investigate the changes of m^5^C methylation in lncRNAs upon IAV infection, Methylated RNA immunoprecipitation sequencing (MeRIP-seq) was performed on H1N1-infected and uninfected A549 cells. We identified 2343 m^5^C peaks on lncRNA transcripts in A549 cells infected with IAV, and 4022 m^5^C peaks in uninfected cells (Fig. 2A). Of the total 5796 identified m^5^C peaks, 1774 were specific to IAV-infected cells, comparing with 3453 were specific to the uninfected control, with 569 sites commonly modified in both groups. These methylated peaks were mapped in 1809 and 2688 annotated lncRNAs in the IAV-infected and control groups, respectively. Among them, 606 m^5^C modified lncRNAs were detected in both groups (Fig. 2A). To evaluate the reliability of our data, LncRNA RPPH1, VTRNA1-1 and SCARNA2, which have been reported harboring m^5^C modification sites [15, 34], were examined and the m^5^C peaks detected in our data were visualized by IGV (Supplementary Fig. 2A in Additional file 4). It has been reported that NSUN2 is the main methyltransferase responsible for the m^5^C methylation of ncRNAs. To validate the specificity of the methylated RNA immunoprecipitation assay used in this study, MeRIP-seq was performed on NSUN2 knockout cells. With 879 m^5^C peaks identified in uninfected cells and 953 peaks in IAV-infected cells, transcriptome-wide mapping of m^5^C revealed a significant reduction in m^5^C modification (Supplementary Fig. 2B in Additional file 4). Notably, the number of m^5^C modification sites in uninfected cells dropped more dramatically than the number in IAV-infected cells. The m^5^C peaks on LncRNA RPPH1, VTRNA1-1 and SCARNA2 identified before did not appear in the NSUN2 knockout cells (Supplementary Fig. 2A in Additional file 4). These results demonstrated that the m^5^C peaks identified by MeRIP-seq are generally reliable.

HOMER software was used to search the consensus motifs in the m^5^C peak regions. Two consensus motifs were identified in the m^5^C peak sites, which were RYCGRH and CCGYUB (Fig. 2B). According to the gene annotation in the reference genome, the distribution of these m^5^C peaks in the whole lncRNA transcriptome was studied. The metagene analysis demonstrated that m^5^C peaks in lncRNAs were obviously enriched around the transcription start site (TSS), and this enrichment was slightly reduced after infection (Fig. 2C).

Classification analysis of the identified m^5^C lncRNAs revealed that the ratios of all the three subtypes of intergenic lncRNAs increased upon H1N1 infection, and the total ratio raised from 15.86 to 35.45%. Accordingly, the proportions for the subtypes of genic lncRNAs decreased in the virus infected cells, and the ratio of m^5^C modified genic lncRNAs decreased from 84.14 to 64.55% (Fig. 2D).

Analysis of the chromosome distribution showed that the m^5^C modified lncRNAs appeared more frequently on chromosomes 1, 2, 17, and 19 than the others, while few modified lncRNAs was located in the Y chromosome (Fig. 2E). Notably, we found that most (about 80%) of the methylated lncRNAs had only one m^5^C peak, whereas about 10% of them contained two peaks and 10% had three or more peaks (Fig. 2F). IAV infection did not significantly change the number of m^5^C modification in each lncRNA and the chromosome distribution of the modified lncRNAs.

### Differentially methylated lncRNAs in IAV infection

**Fig. 3:**
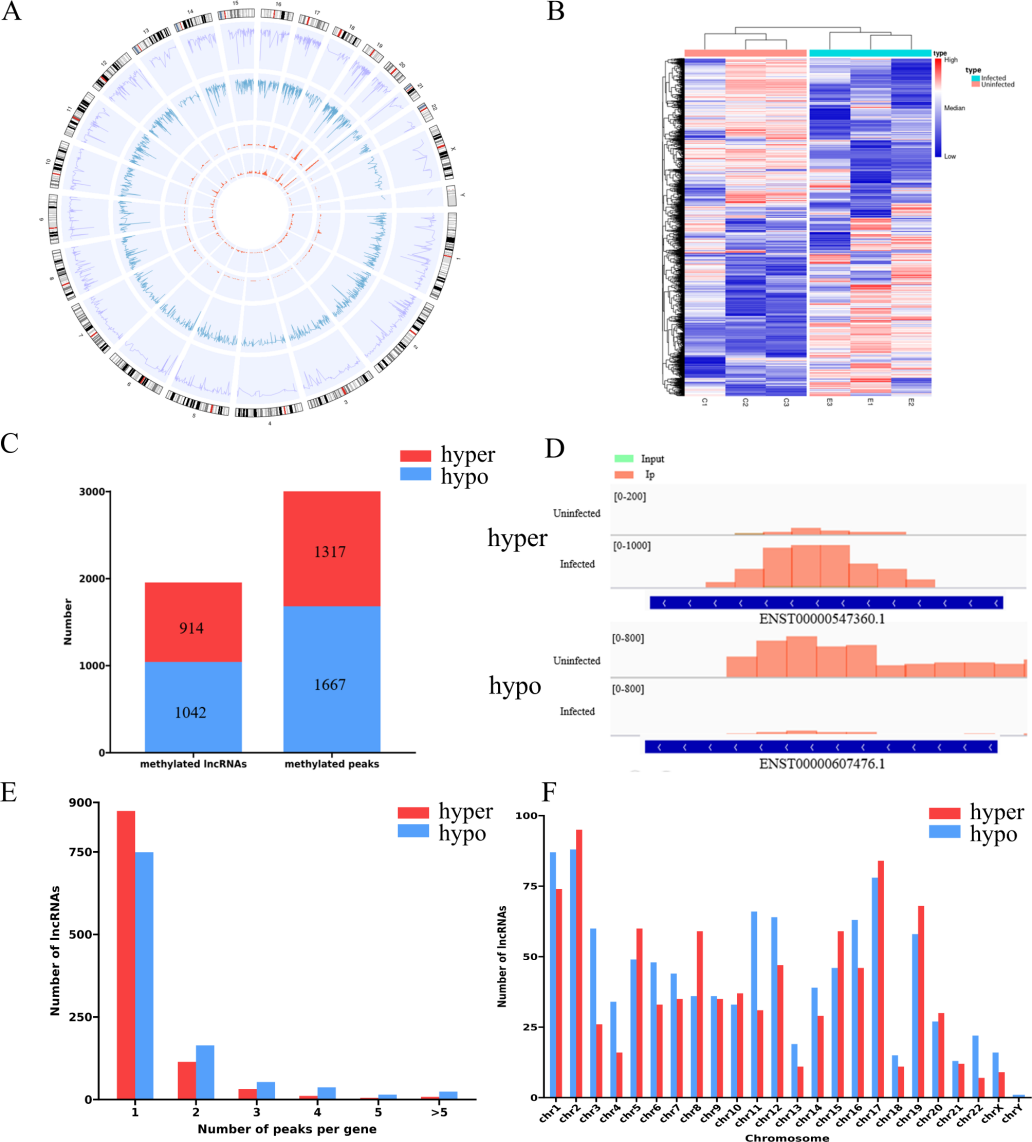
Differentially methylated (DM) lncRNAs in H1N1-infected cells versus uninfected cells. **(A)** Circos plot showing the distribution and density of the m^5^C sites within lncRNAs on each chromosome in uninfected and H1N1-infected cells. From outside to inside, the first lap is the track of hg38 genome, the second and third laps show the abundance of m^5^C modified sites, and the inner two laps show the density of different m^5^C sites on chromosomes. **(B)** Hierarchical clustering analysis showing the different m^5^C modification patterns for lncRNAs between H1N1 infected and uninfected groups. **(C)** Numbers of DM m^5^C peaks and DM lncRNAs. **(D)** Representative DM m^5^C peaks visualized by Integrative Genomics Viewer (IGV). **(E)** The number of DM lncRNAs with different number of m^5^C peaks. **(F)** Distribution of DM lncRNAs on chromosomes

When the distribution of m^5^C methylation sites on the whole genome was analyzed by R-circlize package, it was shown that the distribution and density of m^5^C peaks on each chromosome were very different between the infected and uninfected cells (Fig. 3A). Clustering analysis was performed to compare the m^5^C modifications in the two groups. In the heatmap, the lncRNAs differentially modified in infected cells could be clearly distinguished from those in the control cells (Fig. 3B), demonstrating the significant alteration of m^5^C modification upon IAV infection.

Compared to the uninfected group, there were 2984 differentially modified (DM) m^5^C sites identified on lncRNAs in IAV-infected cells, including 1317 hypermethylated and 1667 hypomethylated sites (Additional file 2). The 2984 differentially methylated m^5^C sites were located across 1956 lncRNAs, of which 914 were hypermethylated and 1042 were hypomethylated (Fig. 3C). The top ten lncRNAs, in which the m^5^C methylation was upregulated or downregulated with the highest fold change values, were respectively listed in Tables 1 and 2. Representative examples of hypermethylated and hypomethylated peaks were visualized by IGV and shown in Fig. 3D. Approximately 83.1% (1623/1956) of the DM lncRNAs contained one DM site, 14.2% (278/1956) had two DM sites and 2.7% had three or more altered methylation peaks (Fig. 3E). Analysis of the distribution of altered m^5^C peaks on chromosomes showed that these alterations were distributed on all chromosomes, with the highest number in chromosome 2 (Fig. 3F).

**Table 1:** Top ten up-methylated lncRNA

PeakChrom	PeakStart	PeakEnd	FoldChange	LncRNA_Transcript_id
Chr12	101,408,458	101,408,858	10.718	ENST00000547360.1
Chr5	179,808,462	179,808,862	10.711	MSTRG.81957.1
chr1	143,735,704	143,736,104	10.619	ENST00000692327.1
Chr3	139,390,382	139,390,782	10.401	ENST00000685754.1
Chr5	173,764,083	173,764,483	10.286	ENST00000671054.1
chr19	43,164,015	43,164,415	10.129	ENST00000635495.1
Chr2	215,297,891	215,298,291	10.05	ENST00000657931.1
Chr5	181,215,936	181,216,336	9.866	ENST00000668115.1
Chr6	26,331,241	26,331,641	9.654	ENST00000655687.1
chr19	4,790,810	4,791,210	9.542	ENST00000598782.2

**Table 2:** Top ten down-methylated lncRNA

PeakChrom	PeakStart	PeakEnd	FoldChange	LncRNA_Transcript_id
Chr6	30,723,631	30,724,031	-11.278	ENST00000607476.1
Chr12	52,245,316	52,245,716	-10.173	ENST00000546686.1
chr19	46,609,148	46,609,548	-9.563	ENST00000597609.1
Chr17	47,682,260	47,682,660	-9.501	ENST00000578482.1
Chr17	7,577,189	7,577,930	-9.475	ENST00000581621.1
Chr3	169,982,600	169,983,364	-8.958	ENST00000479626.1
Chr12	53,443,474	53,443,874	-8.931	ENST00000547717.1
Chr12	122,981,552	122,981,952	-8.891	ENST00000540866.2
Chr17	38,920,624	38,921,024	-8.672	ENST00000580121.1
Chr8	143,580,478	143,580,878	-8.591	ENST00000623257.1

### GO Enrichment and KEGG pathway analyses of lncRNAs harboring DM m^5^C sites

**Fig. 4:**
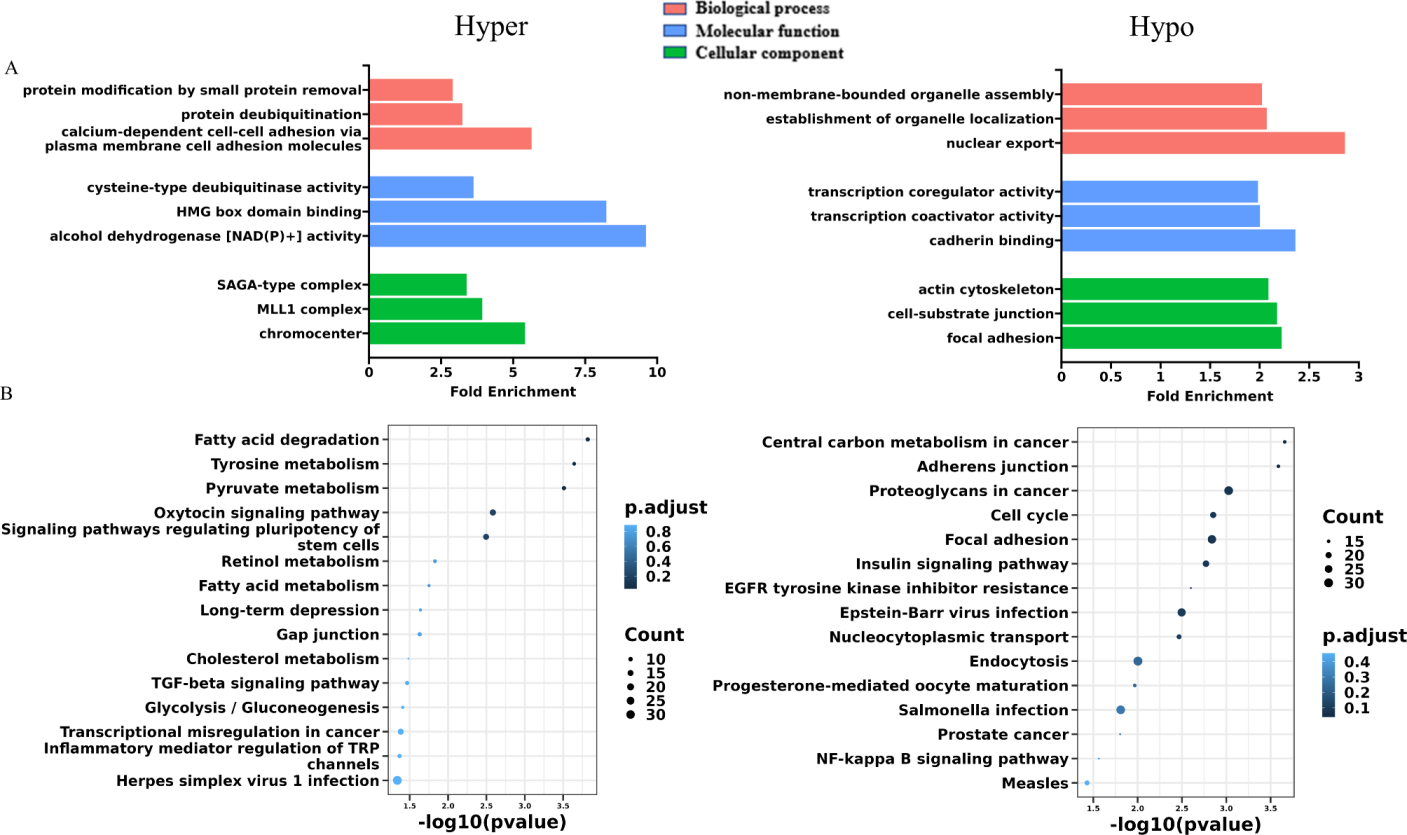
GO enrichment and KEGG pathway analyses of cis-target genes of DM lncRNAs. **(A)** Bar plot showing the top 3 GO terms of biological processes (BP), molecular functions (MF) and cellular components (CC) that were significantly enriched for the genes targeted by DM lncRNAs. **(B)** Dot plot showing the top 15 pathways significantly enriched for the DM lncRNAs.

To explore the potential functions of lncRNA m^5^C methylation in IAV infection, the nearest protein-coding genes paired with the differentially modified lncRNAs were searched out and used for Gene Ontology (GO) enrichment and Kyoto Encyclopedia of Genes and Genomes (KEGG) pathway analyses. GO analysis based on biological processes (BP) revealed that the target-genes of up-methylated lncRNAs (H1N1-infected vs. uninfected cells) were significantly enriched in protein modification by small protein removal, protein deubiquitination, and calcium-dependent cell-cell adhesion via plasma membrane cell adhesion molecules, while the down-methylated genes were largely enriched in non-membrane-bounded organelle assembly, establishment of organelle localization and nuclear export. The molecular functions (MF) output showed that the genes targeted by the up-methylated lncRNAs were notably involved in cysteine-type deubiquitinase, HMG box domain binding and alcohol dehydrogenase (NAD(P)+) activity. In contrast, the down-methylated lncRNAs were predominantly associated with genes playing roles in transcription coregulator activity, transcription coactivator activity and cadherin binding. The cellular components (CC) data showed that up-methylated lncRNAs mainly targeted genes in SAGA-type complex, MLL1 complex and chromocenter, while the down-methylated lncRNAs were primarily associated with the genes in actin cytoskeleton, cell-substrate junction and focal adhesion (Fig. 4A).

The KEGG pathway analysis revealed that some up-methylated lncRNAs were significantly associated with genes involved in metabolism (e.g. Fatty acid metabolism, Tyrosine metabolism, Pyruvate metabolism, Retinol metabolism, Cholesterol metabolism). The down-methylated lncRNAs were notably related to genes in Central carbon metabolism and Adherens junction. A number of target genes of hypomethylated lncRNAs were involved in some infection related pathways (e.g. Epstein-Barr virus infection, Salmonella infection) (Fig. 4B).

### Conjoint analysis of DM and DE lncRNAs

**Fig. 5:**
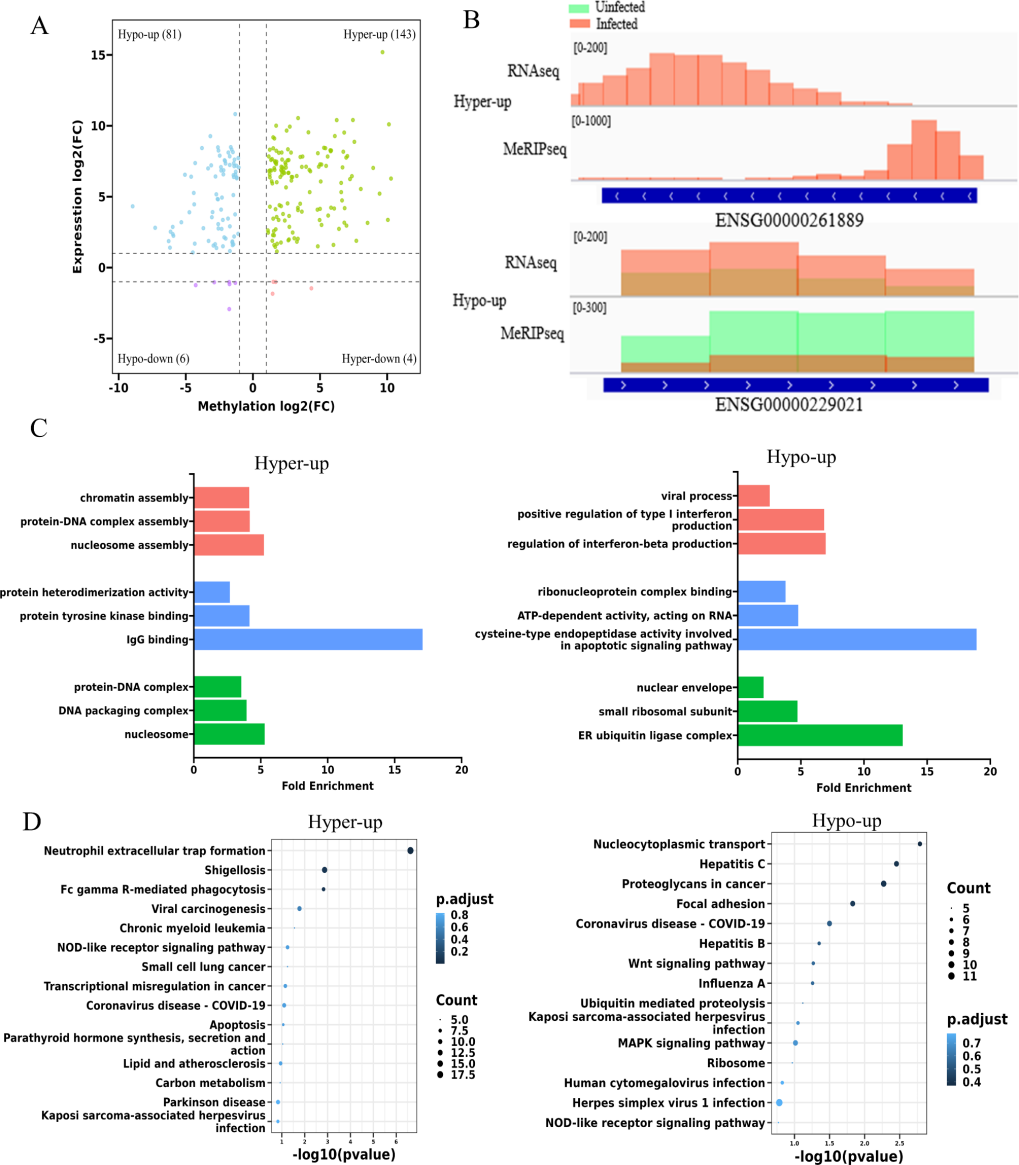
Conjoint analysis of DM and DE lncRNAs. **(A)** Four-quadrant scatterplots showing the distribution of lncRNAs with significant changes in both m^5^C modification and expression levels after H1N1 infection. **(B)** Examples of hyper-up and hypo-up lncRNAs displayed by IGV. **(C)** The top 3 GO terms of biological processes (BP), molecular functions (MF), and cellular components (CC) significantly enriched for the genes targeted by hyper-up and hypo-up lncRNAs. **(D)** The top 15 significantly enriched pathways for the genes targeted by hyper-up and hypo-up lncRNAs.

Conjoint analysis was then performed to explore the relationship between m^5^C epitranscriptomic modification and lncRNA expression level. Conjoint analysis of the DM and DE lncRNAs identified 143 hyper-up genes with both m^5^C modification and expression levels up-regulated, 4 hyper-down lncRNAs with up-regulated modification and decreased expression levels, 81 hypo-up lncRNAs with down-regulated modification and increased expression levels, and 6 hypo-down lncRNAs with down-regulated modification and decreased expression levels (fold change > 2, P-value < 0.05) (Fig. 5A and Additional file 3). An example of a hyper-up lncRNA and a hypo-up lncRNA visualized by IGV were presented in Fig. 5B.

GO and KEGG analyses of the genes targeted by the hyper-up and hypo-up lncRNAs were performed to investigate the potential roles of these lncRNAs in IAV infection. For the hyper-up lncRNAs, BP analysis showed that they are mainly participated in chromatin assembly, protein-DNA assembly and nucleosome assembly. MF data revealed the involvement of these lncRNAs in IgG binding, protein tyrosine kinase binding and protein heterodimerization activity. For CC, the target genes were mainly enriched in protein-DNA complex, DNA packaging complex and nucleosome. As for hypo-up lncRNAs, viral process and regulation of interferon production pathways were highlighted by BP analysis. For MF, target genes were enriched in cysteine-type endopeptidase activity involved in apoptotic signaling pathway, ATP-dependent activity and ribonucleoprotein complex binding. The CC analysis found that genes targeted by hypo-up lncRNAs were mainly enriched in ER ubiquitin ligase complex, nuclear envelope and small ribosomal subunit (Fig. 5C). KEGG pathway analyses highlighted the association of hyper-up lncRNAs with Neutrophil extracellular trap formation, and the link of hypo-up lncRNAs with Nucleocytoplasmic transport and Hepatitis C pathway. It is worth noting that most of the genes targeted by these lncRNAs were involved in pathogen recognition and disease process, and both hyper-up and hypo-up lncRNAs targeted genes in NOD-like receptor signaling pathway and Coronavirus disease - COVID-19 pathway (Fig. 5D). These data implied the potential regulation roles of the DM and DE lncRNAs in IAV infection and pathogenesis.

**Fig. 6:**
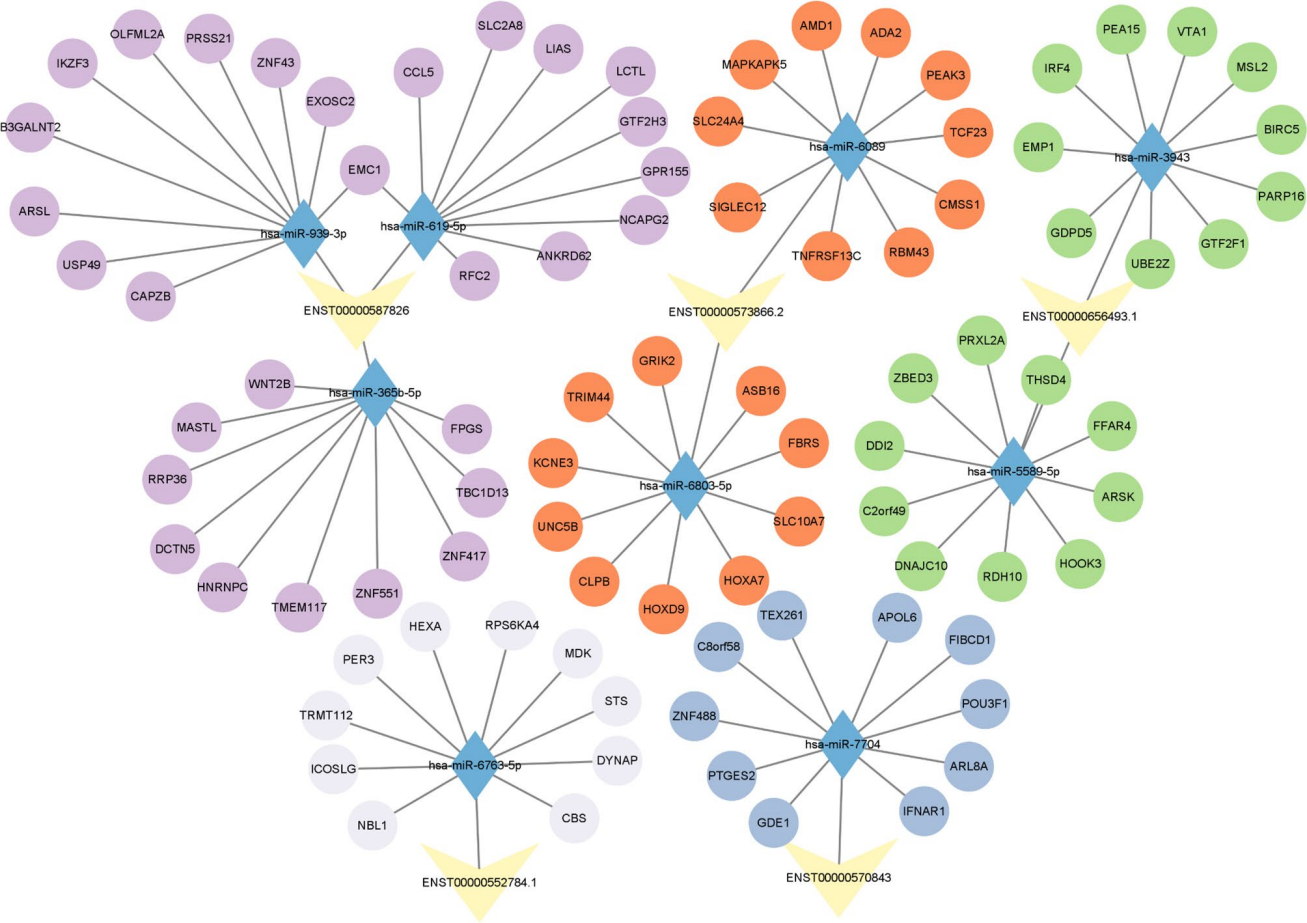
LncRNA-miRNA-mRNA interaction network. The yellow triangles indicate lncRNAs. The blue diamonds represent miRNAs. The circular discs stand for mRNAs regulated by the linked miRNAs.

Researches have demonstrated that lncRNAs can act as microRNA (miRNA) sponges to regulate the expression level of protein-coding genes. To explore the possible regulatory roles of the DM and DE lncRNAs in IAV infection, five lncRNAs including three hyper-up lncRNAs (ENST00000587826.1, ENST00000656493.1, ENST00000570843.1) and two hypo-up lncRNAs (ENST00000573866.2, ENST00000552784.1) were picked out by retrieving lncbook and miRwalk databases, and their competing endogenous RNA (ceRNA) network were constructed. As shown in Fig. 6, the 5 lncRNAs were found to associate with 9 miRNAs, which could regulate the transcription of dozens of mRNAs that playing distinct roles in cell biology.

## Discussion

It has been reported that IAV infection alters the expression of numerous host lncRNAs, suggesting that this class of noncoding RNAs may play important regulation roles in the virus-host interaction [22–24]. Previous reports have revealed that some lncRNAs, such as TSPOAP1-AS1 [30], lnc-MxA [35] and lnc-Lsm3b [36], are upregulated in response to IAV infection and they promote virus replication by suppressing the production of type I interferon. LncRNA PAAN [32] and IPAN [33] have been shown to be induced to promote viral RNA synthesis by association with viral PA and PB1 proteins, respectively. Lnc45 was found to be highly upregulated by different IAV subtypes, and it suppressed influenza virus replication by inhibiting viral polymerase activity and retaining NP and PA in the nucleus of infected cells through its stem-loop arm [37]. The expression of LncRNA ACOD1 is elevated during IAV infection to facilitate virus replication by modulating cellular metabolism [38]. LncRNA NRAV could promote IAV replication by suppressing the initial transcription of several key ISGs, including IFIT2, IFIT3, OASL, IFITM3 and MxA, but it is dramatically downregulated during IAV infection [28]. Several IAV-upregulated lncRNAs have been found to inhibit IAV replication. For example, lnc-ISG20 [25] and ISR [29] function as interferon-stimulated genes (ISGs), and LncRNA-155 [26], NEAT1 [39], SAAL [40], RDUR [41], AVAN [42] and IVRPIE [43] can suppress IAV replication by promoting innate immune responses to viral infection. However, it remains unknown how the expression levels of these lncRNAs are regulated and whether RNA modifications play roles in the lncRNA regulation in response to the virus infection.

In this study, we examined the expression of lncRNAs in H1N1-infected cells, and detected more lncRNAs on each chromosome in the infected group than the uninfected group, indicating an overall upregulation of lncRNAs expression after H1N1 infection. The phenomenon that the majority of DE lncRNAs have elevated expression levels upon IAV infection had been observed in H1N1 and H3N2 infected cells [24, 26]. Here, by differential expression analysis, we identified 1566 DE lncRNAs including 1327 upregulated and 239 downregulated ones in H1N1-infected A549 cells. It is worth exploring how the abundance of these lncRNAs are regulated and what roles they play in IAV infection.

In RNA metabolism, m^5^C has been reported to act as a modulator for the stability [44], nuclear export [8] and protein translation [9] of cellular mRNAs. Studies have also found that m^5^C modification of enhancer RNA [45] and lncRNA [46] can increase the stability of these noncoding RNAs. m^5^C and m^6^A modifications of LncRNA NKILA have been found to facilitate cholangiocarcinoma growth and metastasis through the miR-582-3p-YAP1 axis [47]. Recently, it was reported that m^5^C methylation in lncRNAs played an important role in the regulation of type I interferons and antiviral innate immunity [15].

By MeRIP-Seq, we studied how the m^5^C modification in lncRNAs was affected by H1N1 IAV infection. The results obtained in A549 cells showed that the m^5^C modification sites and distribution on lncRNAs were globally altered after IAV infection. A total of 1317 upregulated and 1667 downregulated m^5^C peaks were detected upon H1N1 infection. GO analysis showed that the genes targeted by the m^5^C differentially modified (m^5^C-DM) lncRNAs in IAV infection were notably enriched in biological processes of calcium-dependent cell-cell adhesion via plasma membrane cell adhesion molecules, protein deubiquitination, nuclear export and organelle localization. KEGG pathway analyses suggested that these m^5^C-DM lncRNAs were mainly associated with metabolism (such as fatty acid metabolism, and tyrosine metabolism) and some infectious pathogens related pathways which were known to be important for virus infections. These data indicated that m^5^C modification on lncRNAs could play important roles in the host responses to IAV infection.

By conjoint analysis of the epitranscriptomic profile and expression profile, we identified 143 ‘hyper-up’, 4 ‘hyper-down’, 81 ‘hypo-up’, and 6 ‘hypo-down’ lncRNAs. GO analysis highlighted the enrichment of target genes of DM and DE lncRNAs in nucleosome assembly, and regulation of type I interferon production pathway which is consistent with the recent report of the involvement of m^5^C methylation in the regulation of lncRNAs expression and type I interferon production [15]. KEGG pathway analyses revealed that these DM and DE lncRNAs were predominantly associated with pathogen recognition and pathogenesis pathways. These results suggest that m^5^C modifications may be involved in multiple pathways to regulate IAV replication by modulating the expression and/or stability of lncRNAs.

Different influenza viruses may influence the host cells in altered pathways or in different degrees. The same virus may also behave differently in varied cells. Our data in this study demonstrated the association of the expression and m^5^C modification with IAV replication. It remains elusive which of these DE and DM lncRNAs identified are common for IAV infection and which of them specifically respond to H1N1 infection in A549 cells. Furthermore, the samples subjected to MeRIP-seq analyses were harvested at 36 hpi. Part of the DE and DM lncRNAs could be indirectly regulated by the cellular factors altered in early infection. Validation of the altered expression and modification of lncRNAs in early infection could be helpful for screening out the key players in IAV infection. Further studies are also needed to answer some key questions on how IAV induces the differential modification level and distribution of m^5^C, what are the roles of the modifications in the regulation of lncRNA expression and function, and what are the functions of m^5^C modification of lncRNAs in IAV replication and pathogenesis.

Recent studies have revealed that the viral genomic and messenger RNAs of retroviruses including HIV-1 and Murine Leukemia Virus are heavily modified with m^5^C, and the modification plays positive roles in ribosomal recruitment and RNA splicing to benefit viral gene expression and virus replication [14, 48]. Post-transcriptional modification analyses performed by Mass spectrometry have found that viral RNAs from positive-sense RNA viruses such as Zika virus, Dengue virus, hepatitis C virus and poliovirus bear m^5^C modification as well as HIV-1 [49]. For IAV, m^6^A modifications on its mRNAs and vRNAs have been mapped. The modification has been shown to be able to increase viral RNA expression, and IAV HA m^6^A mutants show reduced pathogenicity in mice [50]. It will be important to map m^5^C sites on IAV transcripts and determine whether the modifications are involved in virus replication and pathogenesis.

## Conclusions

In conclusion, we performed a transcriptome-wide 5-methylcytosine modification analysis of lncRNAs in A549 cells infected with influenza A virus, and demonstrated the significant alteration of m^5^C modification upon IAV infection, suggesting a link between lncRNA m^5^C modification and IAV infection. The data obtained in this study could provide new insights into our understanding of virus-host interactions in influenza virus infection, and prompt further studies to explore the potential of lncRNAs as diagnostic markers and therapeutic targets for the virus infection caused diseases.

## Methods

### Cell culture and virus infection

Human alveolar basal epithelial adenocarcinoma cell line (A549) was purchased from Shanghai Institute of Biochemistry and Cell Biology, Chinese Academy of Sciences. Influenza virus isolate A/WSN/33 (H1N1) was donated by Dr. Long Liu. A549 cells were cultured in Ham’s F-12 K (BasalMedia) containing 10% fetal bovine serum (FBS, Hyclone), 100 U/ml penicillin and 100 µg/ml streptomycin (Sangon Biotech) at 37℃ in 5% CO_2_. A549 cells were seeded in 100 mm cell culture dishes. When the confluence reached 80–90%, the cells were infected with influenza A virus strain A/WSN/33 at an MOI of 0.1, incubated at 37℃ for 2 h, and then the media was replaced with F-12 K supplemented with 1% bovine serum albumin (BSA, Solarbio), antibiotics and TPCK-treated Trypsin (Sigma). Cell samples were harvested at 36 hpi for subsequent total RNA extraction. All samples are analyzed in triplicate.

### RNA extraction and fragmentation

Three repeats of IAV-infected and uninfected samples were obtained. RNA was extracted from cells using RNAprep pure Cell / Bacteria Kit (TIANGEN) following the manufacturer’s instructions. Denaturing agarose gel electrophoresis was used to measure RNA integrity and gDNA contamination (Supplementary Fig. 3 in Additional file 4). The concentration of total RNA was determined by NanoDrop ND-1000 spectrophotometer (Thermo Fisher Scientific). The quality of RNA was assessed by the ratio of OD_260_/OD_280_, and the samples with the value between 1.8 and 2.1 were marked as qualified (Supplementary Table 1 in Additional file 4).

### RNA Library construction and sequencing

**Fig. 7:**
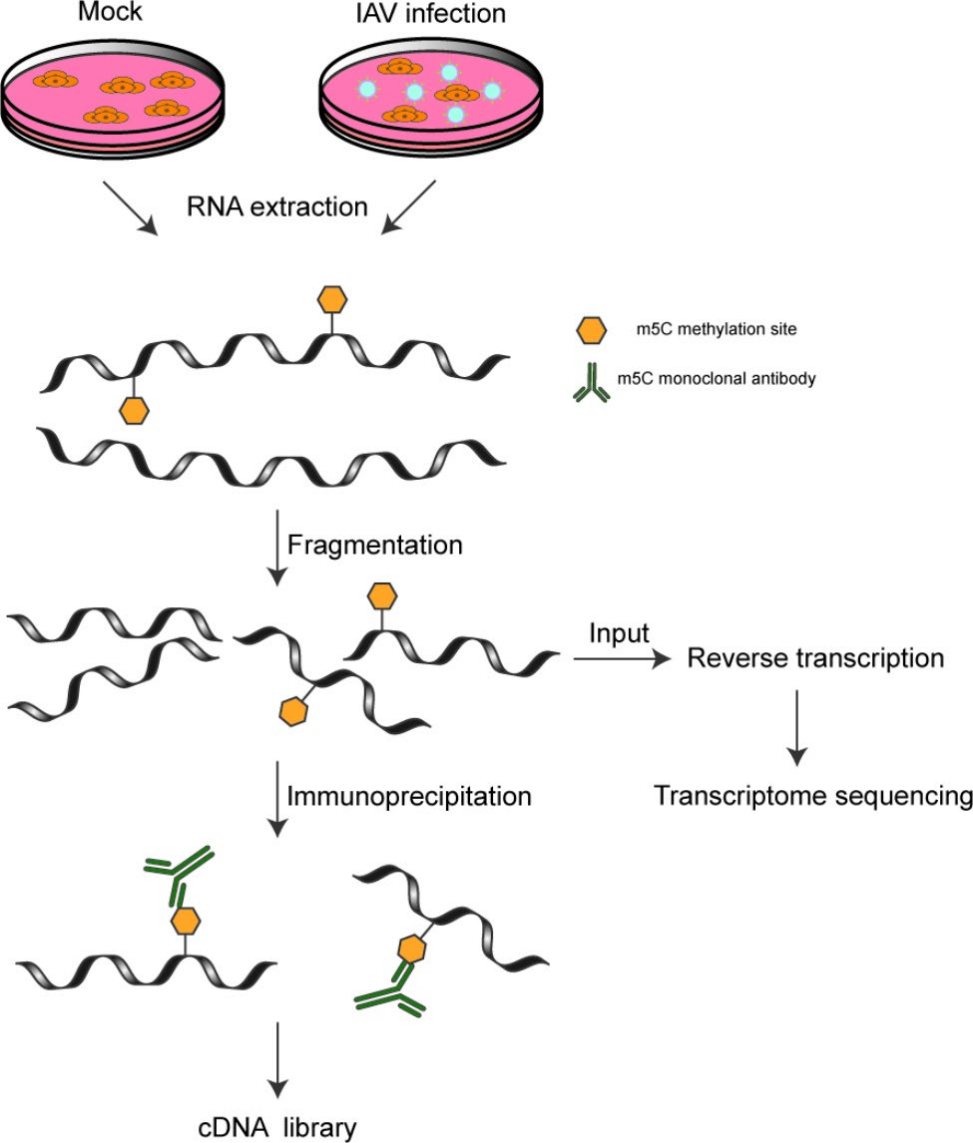
Flowchart illustrating the construction of cDNA libraries for m^5^C-modified lncRNA transcriptome sequencing of uninfected and H1N1-infected A549 cells. A549 cells were infected with H1N1 at an MOI of 0.1. RNA was extracted at 36 hpi. After RNA fragmentation, RNA was divided into two aliquots. One aliquot was analyzed by RNA sequencing to identify the input lncRNAs. The other aliquot was used for MeRIP-seq assay to enrich and identify m^5^C modified lncRNAs.

Transcriptome high throughput sequencing was performed by DIATRE Biotech (Shanghai, China). Briefly, rRNAs were removed from the total RNA with NEBNext® rRNA Depletion Kit (New England Biolabs, Inc., Massachusetts, USA). MeRIP-Seq and RNA-Seq were performed by DIATREBiotech Inc. (Shanghai, China). Immunoprecipitation of m^5^C RNA was performed with the GenSeqTM m^5^C RNA IP Kit (GenSeq Inc) by following the manufacturer’s instructions. Briefly, fragmented RNA was incubated with anti-m^5^C polyclonal antibody (ABclonal) in IPP buffer for 1 h at 4℃. The mixture was then immunoprecipitated by incubation with protein-A beads (Thermo Fisher) at 4℃ for an additional 2 h. Then, bound RNA was eluted from the beads with Proteinase K for 30 min at 55℃, and purified by RNA clean&concentrantorTM-5 (ZYMO Research). RNA-seq libraries for the input and IP RNA samples were generated using NEBNext® Ultra II Directional RNA Library Prep Kit (New England Biolabs). The library quality was evaluated with BioAnalyzer 2100 system (Agilent Technologies) (Supplementary Tables 2 and Supplementary Fig. 4 in Additional file 4). Library sequencing was performed on an illumina NovaSeq 6000 instrument with 150 bp paired-end reads. The sample preparation processes are illustrated in the flowchart in Fig. 7.

### Quality control and transcriptome assembly

Quality control of the paired-end reads was performed with Q30, which was followed by trimming of the 3’ adaptor and removal of low-quality reads using Cutadapt software (v1.9.3) [51]. After that, clean reads of all libraries were aligned to the reference genome (Homo sapiens.GRCh38) by Hisat2 software (v2.0.4) [52]. Then, input data reads were aligned to the reference genome and used for transcriptome assembly and quantification by Stringtie (v2.2.1) [53]. Novel transcripts were annotated using GffCompare software (v0.11.2) [54].

### Identification and quantification of lncRNAs

Within the annotated transcripts, lncRNAs were filtered by the classcodes of x (exonic overlap with reference on the opposite strand), u (intergenic transcripts), i (transcripts entirely within intron), j (at least one splice junction is shared with a reference transcript), and o (other same strand overlap with reference exons). Transcripts with length > 200 bp and exon number > 2 were maintained. The coding potential of these transcripts was predicted by CNCI (v2) [55], CPC2 [56], CPAT (v3.0.0) [57] and PLEK (v1.2) [58] software, and transcripts with coding potential were removed. After these steps, identified lncRNAs were used for quantification analysis by FeatureCounts (v2.0.1) [59]. Deseq2 software was then applied to search differentially expressed lncRNAs (|FC|>2, pvalue < 0.05) [60]. The distribution of lncRNA on gene was analyzed by FEELnc [61].

### MeRIP-seq data analysis

After transcriptome assembly, annotated lncRNA transcripts were used for m^5^C peak calling and lncRNA quantification. Methylated sites of lncRNAs (peaks) were identified by MACS2 software [62]. Differentially methylated sites were determined using DiffBind software [63]: m^5^C peaks with a log2FC > 1 (P-value < 0.05) in infected cells were considered to be up-regulated, and those with log2FC < -1 were down-regulated.

### Bioinformatics analysis

The distribution of lncRNA m^5^C peaks on chromosome was analyzed by R package Circlize [64]. The normalized read counts were used for clustering analysis by R package Pheatmap [65]. Guitar package was used for analyzing the distribution of m^5^C peaks on lncRNAs [66]. The read alignments on genome were visualized using the interactive analysis tool Integrative Genomics Viewer (IGV) [67]. HOMER software was used to search the motifs in the m^5^C peak regions. The Gene Ontology (GO) and Kyoto Encyclopedia of Genes and Genomes (KEGG) [68] analyses were performed based on the cis-target genes of lncRNAs to explore the functions of these lncRNAs by R package ClusterProfiler [69]. The interactions between lncRNAs and miRNAs were retrieved from lncbook (https://ngdc.cncb.ac.cn/lncbook/home) [70], and the association between miRNA and mRNA was predicted by miRWalk (http://mirwalk.umm.uni-heidelberg.de/) [71]. The lncRNA-miRNA-mRNA network was constructed by Cytoscape based on the interactions of lncRNAs, miRNAs and mRNAs.

### Quantitative real-time PCR

Total RNAs from H1N1-infected and uninfected A549 cells were used to synthesize cDNA using the HiFiScript gDNA Removal cDNA Synthesis Kit (Cwbio, China). Quantitative Real-time PCR (qPCR) was carried out using ChamQ Universal SYBR qPCR Master Mix (Vazyme, China) according to the manufacturer’s instructions. GAPDH was used as a normalization control. The relative expression level of each lncRNA was calculated with the 2^−ΔΔCt^ method. All samples were analyzed in triplicate. The primer sequences used in the qPCR are listed in Table 3.

**Table 3:** Primer sequences used for quantitative RT-PCR

Gene_id	Primer sequences-F(5′ -3′)	Primer sequences-R(5′ -3′)
GAPDH	CTACACTGAGCACCAGGTGG	CATGAGGTCCACCACCCTG
ENSG00000204709	GACTCAATTGCCTTCGCAGC	TACAAGGCAGGGGCCAATTC
ENSG00000243819	GAGGCTTTCGAGTCTCTGCC	CTTCCACTCGGAATTCGCAC
ENSG00000281100	GCAGATGGACAGGGCTTGTA	CTTAGGCCACAGGAGTGATCC
ENSG00000272666.1	TCACATAACCTGTGGCAAGC	TGCAAAACGTGCTGTTAGTAAGG
ENSG00000255101.1	AAGGAGATCATGAATGCGGGC	GTATTGACCACAAGTCCCACCT
MSTRG.82,485	ACTCTCTTTGCCATGCTGTT	TCTGGCCAACAATGTCACAA
ENSG00000261889	TGTCGGATGGATTAGACTTGC	GCGTTTGCCTGACCAACAAT
ENSG00000277511	TTCAATAAGGCAGCGGGACG	CTGGGACTTTCAGGGTGGAG
ENSG00000278709	AACCAAACCTACCCACAACG	ACCACTAAGTCAATCCCAGGTG
ENSG00000225964	CCACCCCCACGAAGAAATTATATATC	GTTAGAGGTGTCTGCTGCAATAATC
ENSG00000258474	TGACAGTGGATTTGTCACCCA	ACTTGGCAGGTCAGTGAAAGG
ENSG00000271851	TGGCCCCAACGTGAATTGTT	TCTTGGCAGTCCAGTAACACA
ENSG00000272335	ACATGGTCAGTATCCTCTCACT	CACTGTCTCACCCCAATGCT
ENSG00000275769	AGGCAGAGGATCAGAGACCT	TCAAAGTCCCCAGAAACAGTGA
ENSG00000278730.1	CTGAAGAAGAATGTCAAGTGGGG	AAGCAATCCTGTCTTTGTGTGG
ENSG00000263731.1	GACTGGCCAAGCATTTGGTG	TCTTGGTACTTTCATGGCTTTATTT
ENSG00000260804	GTGAATATGAAACAAGCTGCAAG	AAGACACTGACCACATGGACTC
ENSG00000273033	AGAGGACAGGGTCGTCTCTT	CTTGCTCAGGCATATGGGGT
ENSG00000279117	AAAATGGGGCTAGTCCAGGC	TGAGGCACCCACCTCTCATA
ENSG00000269044.2	CCACCATCAGGATTTTGGAGA	GTCAAGCGCTGGGATTGTTC

## Electronic supplementary material

Below is the link to the electronic supplementary material.

Supplementary Material 1

Supplementary Material 2

Supplementary Material 3

Supplementary Material 4

## Data Availability

The raw sequence data reported in this paper have been deposited in the Genome Sequence Archive in BIG Data Center, Beijing Institute of Genomics (BIG), Chinese Academy of Sciences, under accession number HRA003296 which is publicly accessible at https://ngdc.cncb.ac.cn/gsa-human. For editors and reviewers, please access the sequence data from https://ngdc.cncb.ac.cn/gsa-human/s/1d1Ht4xw. All other data supporting the findings of this study are available from the corresponding author on reasonable request.

## Abbreviations

IAV: Influenza A virus
A549: Human alveolar basal epithelial adenocarcinoma cell line
lncRNAs: Long non-coding RNAs
miRNA: microRNA
mRNA: Messenger RNA
m^5^C: 5-methylcytosine
MeRIP-Seq: Methylated RNA immunoprecipitation sequencing
RNA-seq: RNA sequencing
HA: Hemagglutinin
ISGs: Interferon-stimulated genes
GO: Gene ontology
BP: Biological processes
CC: Cellular components
MF: Molecular functions
KEGG: Kyoto Encyclopedia of Genes and Genomes
FBS: Fetal bovine serum
BSA: Bovine serum albumin
PBS: Phosphate buffered saline
FE: Fold enrichment
GAPDH: Glyceraldehyde-3-phosphate dehydrogenase
DM: Differentially modified
DE: Differentially expressed
