# Supplementary material for: Transcriptome-wide 5-methylcytosine modification profiling of long non-coding RNAs in A549 cells infected with H1N1 influenza A virus
Source: BMC Genomics. 2023 Jun 12;24:316. doi: 10.1186/s12864-023-09432-z (PMC10258786; doi:10.1186/s12864-023-09432-z)
Supplement: Supplementary file 4 — Supplementary Material 4 [file 12864_2023_9432_MOESM4_ESM.docx]

**Supplementary Information**

**Transcriptome-wide 5-methylcytosine Modification Profiling of Long Non-coding RNAs in A549 Cells Infected with H1N1 Influenza A Virus**

**Authors:** Shengqiang Jiang^a#^, Jing Hu^a#^, Yang Bai^a^, Ruiwei Hao^a^, Long Liu^b^, Hongying Chen^a*^

**Affiliations:**

^a^ College of Life Sciences, Northwest A&F University, Yangling, Shaanxi, 712100, P. R. China

^b^ School of Basic Medical Sciences, Hubei University of Medicine, Shiyan, 442000，P. R. China,

* Corresponding author: chenhy@nwsuaf.edu.cn (H.C.). Tel: +86- 15339264582 (H.C.).

**Supplementary Notes included:**

1. Supplementary Figure 1: (A) Classification of DE lncRNAs by FEELnc. (B) Classification of lncRNAs by FEELnc in uninfected and H1N1-infected cells
2. Supplementary Figure 2: (A) Known m^5^C modification sites in lncRNAs displayed by IGV from our data. (B) Number of m^5^C peaks identified in different cell lines before and after influenza virus infection. (C) Validation of the knockout of NSUN2 gene in the N2KO cell line by Sanger sequencing.
3. Supplementary Figure 3: Agarose gel electrophoresis examination of RNA samples extracted from A549 cells
4. Supplementary Figure 4: Examination of the library quality by Bioanalyzer 2100 (Agilent Technologies)
5. Supplementary Table 1: RNA quantification and quality measured by NanoDrop ND-1000
6. Supplementary Table 2: Sequencing library quality examined by Agilent Bioanalyzer 2100


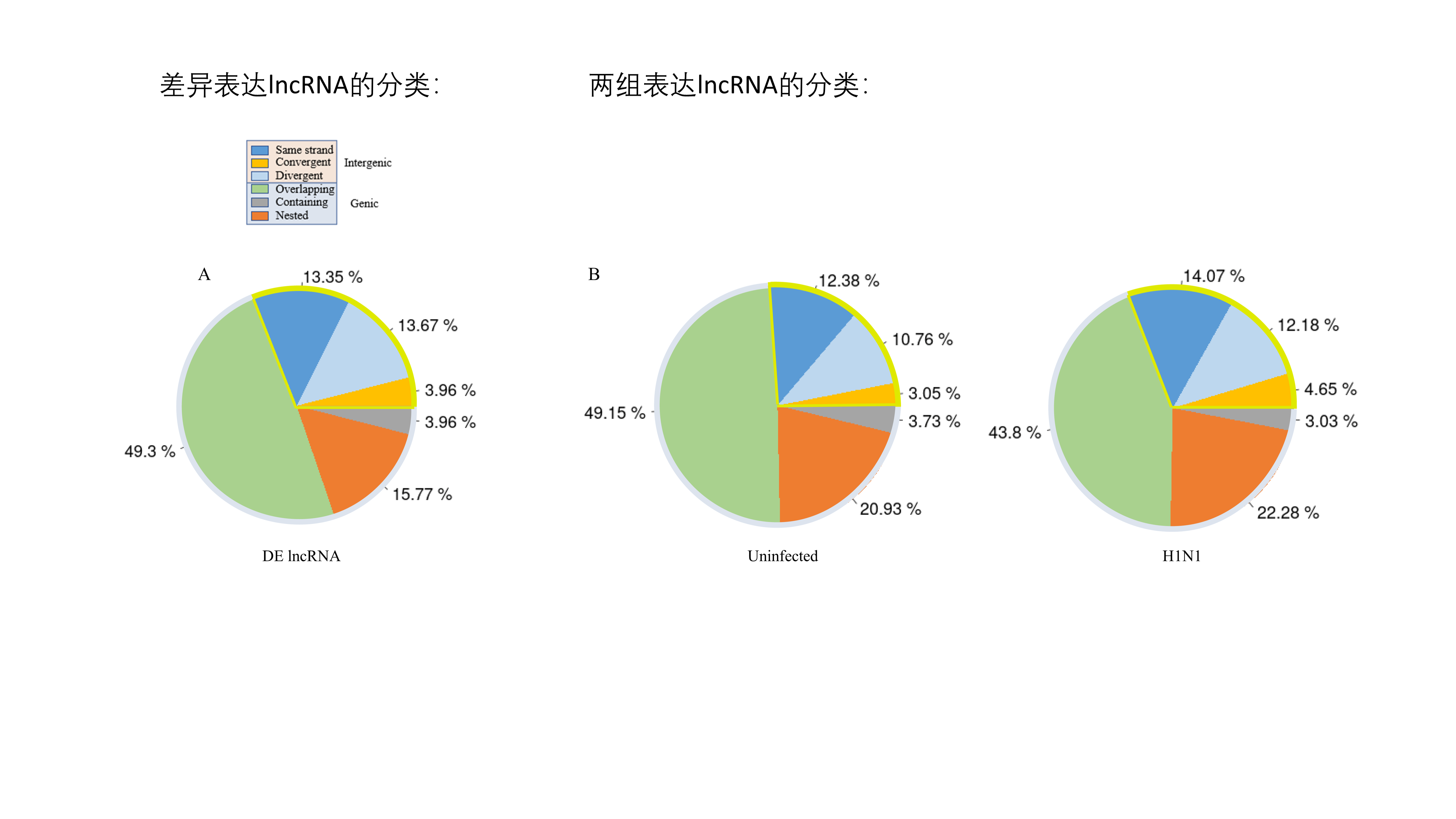


**Supplementary Figure 1: (A) Classification of DE lncRNAs by FEELnc. (B) Classification of lncRNAs by FEELnc in uninfected and H1N1-infected cells**

**
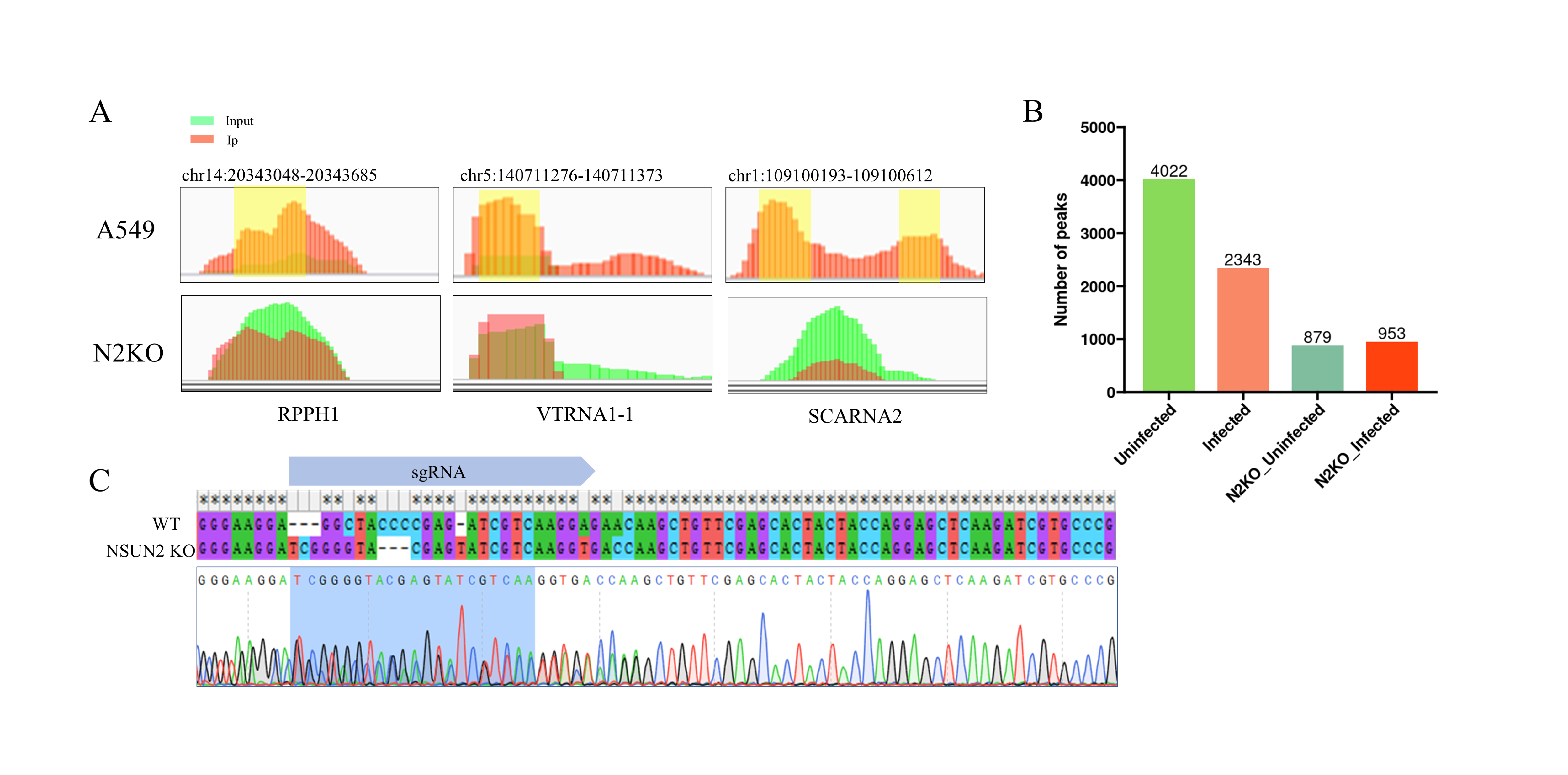
**

**Supplementary Figure 2:** **(A) Known m^5^C modification sites in lncRNAs displayed by IGV from our data. (B) Number of m^5^C peaks identified in different cell lines before and after influenza virus infection. (C) Validation of the knockout of NSUN2 gene in the N2KO cell line by Sanger sequencing.**

**Note:** NSUN2 knockout A549 (N2KO) cell line was generated by targeting the second exon of the NSUN2 gene using the CRISPR method. RPPH1 in chr14:20343234, VTRNA1-1 in chr5:140711276-140711373, and SCARNA2 in chr1:109100193-109100612 are lncRNAs that have been reported harboring m^5^C modification sites. These lncRNAs exhibit a significant reduction in the m^5^C modification levels in N2KO cells, which is consistent with previous reports. Yellow areas indicate potential modification sites.


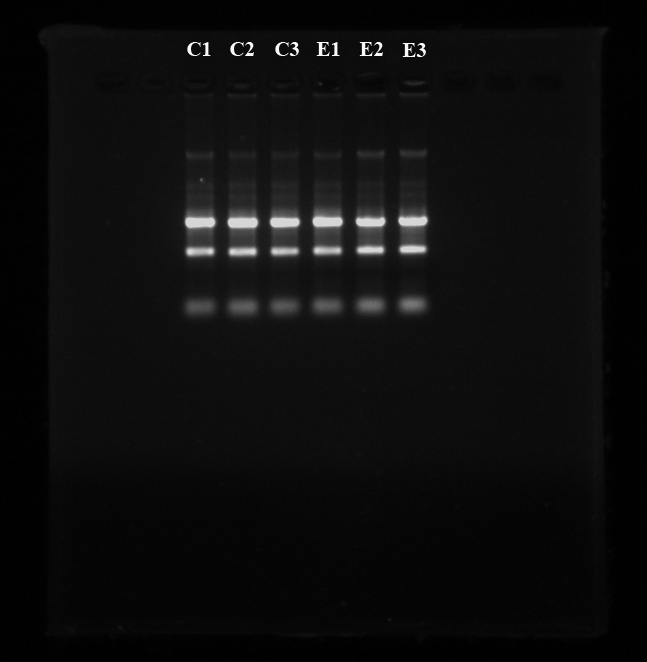


**Supplementary Figure 3 Agarose gel electrophoresis examination of RNA samples extracted from A549 cells.**

**Note：**C1-C3 are RNAs from uninfected cells; E1-E3 are RNA samples from IAV infected cells. A549 cells infected with influenza A virus strain A/WSN/33 at an MOI of 0.1 were harvested at 36 hpi for total RNA extraction.

**
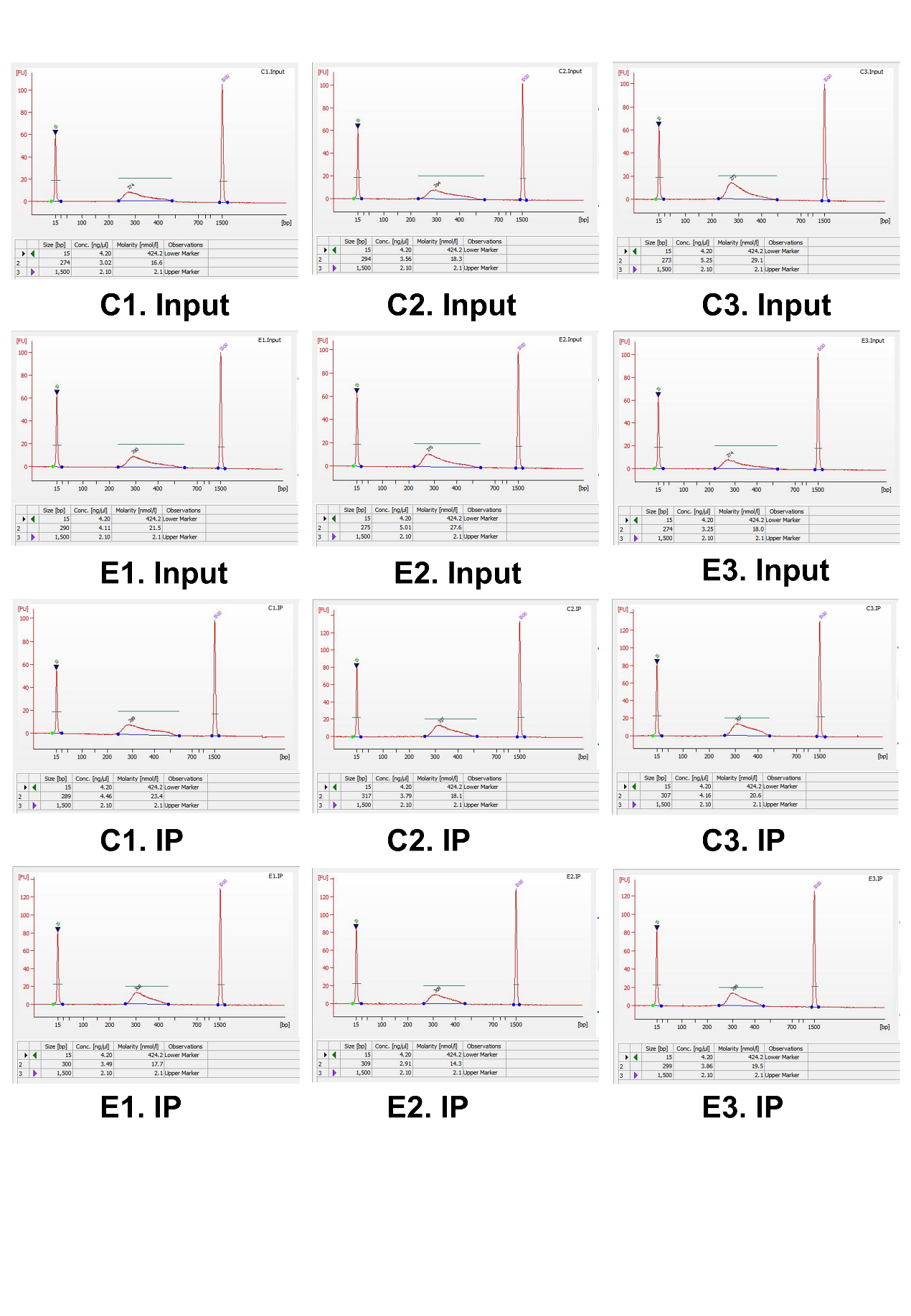
**

**Supplementary Figure 4 Examination of the library quality by Bioanalyzer 2100 (Agilent Technologies)**

**Supplementary Table 1 RNA quantification and quality measured by NanoDrop ND-1000**

| **Sample name** | **OD260/280**  **Ratio** | **OD260/230**  **Ratio** | **Conc. (ng/μl)** | **Volume (μl)** | **Quantity (ug)** | **QC result** |
| --- | --- | --- | --- | --- | --- | --- |
| C1 | 1.91 | 1.42 | 380.22 | 125 | 47.53 | Pass |
| C2 | 1.86 | 1.53 | 388.88 | 125 | 49.78 | Pass |
| C3 | 1.90 | 1.48 | 382.39 | 126 | 48.18 | Pass |
| E1 | 1.93 | 1.02 | 206.70 | 88 | 18.18 | Pass |
| E2 | 1.93 | 0.88 | 231.68 | 82 | 19.00 | Pass |
| E3 | 1.91 | 1.93 | 342.09 | 141 | 48.23 | Pass |

**Supplementary Table 2 Sequencing library quality examined by Agilent Bioanalyzer 2100**

| **Sample name** | **Size**  **(bp)** | **Conc. (ng/μl)** | **Conc. (nmol/μl)** | **Volume (μl)** | **Total Amount**  **(ng)** |
| --- | --- | --- | --- | --- | --- |
| C1. Input | 274 | 3.02 | 16.6 | 10 | 30.2 |
| C2. Input | 294 | 3.56 | 18.3 | 10 | 35.6 |
| C3. Input | 273 | 5.25 | 29.1 | 10 | 52.5 |
| E1. Input | 290 | 4.11 | 21.5 | 10 | 41.1 |
| E2. Input | 275 | 5.01 | 27.6 | 10 | 50.1 |
| E3. Input | 274 | 3.25 | 18.0 | 10 | 32.5 |
| C1. IP | 289 | 4.46 | 23.4 | 10 | 44.6 |
| C2. IP | 317 | 3.79 | 18.1 | 10 | 37.9 |
| C3. IP | 307 | 4.16 | 20.6 | 10 | 41.6 |
| E1. IP | 300 | 3.49 | 17.7 | 10 | 34.9 |
| E2. IP | 309 | 2.91 | 14.3 | 10 | 29.1 |
| E3. IP | 299 | 3.86 | 19.5 | 10 | 38.6 |
